# Mr. Sheldrake, on Distortions of the Feet

**Published:** 1801-05

**Authors:** T. Sheldrake

**Affiliations:** Strand


### *To the Editors of the Medical and Physical Journal.*

GENTLEMEN,

IN May, 1800, J. Haythorne, Esq. of Bristol, consulted me about his daughter, of whom he gave the following account.

She had been healthy from her birth till about the age of two years, when she had a fever, and at the same time lost the use of all her limbs by (as it was supposed) a paralytic stroke. From this she recovered; but it was soon observed that the right foot was weaker than the other. As she grew, this defect continued to increase; the foot became weaker, and deformed to such a degree that at last she had no power to use it; at least, it had no capacity for voluntary motion: it became rigidly fixed in its deformed state, with much tendency to increase in deformity, in consequence of the very imperfect manner in which she was able to walk about. The left leg and foot began to assume the same appearances, though in a less degree; electricity, the Bath waters, &c. and every means that could be devised to obviate or diminish this defect, had been tried without effect, for it increased so much that at last she could not walk across the room without being led by an assistant and supported by a stick; she began to grow distorted from the pelvis upwards; and as every circumstance of the disease was rapidly increasing, without any well-founded expectation of restoring the form and powers of the parts, application was made to me, to contrive some means of preventing her from getting worse, and, if possible, of enabling her to move about in the situation she then was.

At this time the right foot was, (as supposed) in consequence of permanent contraction of the tendo achillis, so drawn downwards that the ball of the great toe, the heel, and the calf of the leg were in a direct line with each other; the foot, by the apparent contraction of the plantar aponeurosis, was much shortened, and rendered hollow on the bottom and the inside; the great toe was drawn up and permanently shortened; the circular head of the astragalus was out of its natural situation, and

---

and lay in a direct line with the front of the leg; the toes were turned inwards, fo far, that when fhe attempted to ftand or walk, fhe refted upon the nails of the three middle toes, as the great toe did not touch the ground: In confequence of this ftate of the foot, every attempt fhe made to walk or ftand upon it tended to increafe the deformity; the leg was flaccid, much wafted, and fhe was not confcious of any degree of fenfibility either in that or the foot. At this time I had a caft in plafter of Paris made from the foot, in prefence of Mr. H. who obferved, that the workman placed the foot as near the natural pofition as it was poffible to be in when he made the mould; from this caft I made four views, in different directions, which are in the annexed Plate 5, and will give a correct idea of the ftate of the foot at the time.

The left foot had made fo much progrefs towards the fame ftate, that, in any other patient, it would have excited confiderable alarm; but in this was fcarcely confidered, or at moft only as fecondary in point of confequence to the other. Both the knees bent inwards, and the diftortion that was beginning to take place above the pelvis, had as yet produced no permanent deformity; and as it was merely the effect of peculiar pofitions occafioned by the deranged action of the legs, it was at this time not an object of attention.

Mr. H. when he applied to me, had no expectations of obtaining more than palliative relief; but, in confequence of my opinion, was induced to place her under my care, to fee what could be effected towards obtaining a cure. Before the end of the firft week that fhe was under my care, fhe felt a warm fenfation pervade the whole of her right leg, which till then had not evinced the leaft fenfibility; and this fenfation became fo permanent, that much attention was requifite, during the progrefs of the cure, to prevent it from becoming exceffive; the form of the foot foon underwent a material change, and continued to do fo progreffively till laft December, when fhe was able to ftand flat on the fole of her foot; and while ftanding upon it, there was little in its appearance to diftinguifh it from the foot of any other perfon.

At this time I had a fecond caft made from the foot, and from this caft I have made the four views reprefented in the annexed Plate 6, viz. Fig. 1, a profile view of the infide of the leg and foot; Fig. 2, a front view of the fame; Fig. 3, a profile view of the outfide of the leg and foot; and Fig. 4, a back view of the fame. In making the drawings reprefented in Plate 5, I placed the LEG in the fame fituations as the leg in Plate 6, confequently, by comparing the feet in each plate with thofe marked with correfponding numbers in the other, a correct opinion may be formed of the alteration produced in the form and appearance of the foot at the different periods when the caſts were taken.

A circumſtance at leaſt as remarkable as the alteration in point of form, is the increaſe of ſize in the whole limb that has taken place during the time that has been employed in the cure; partly by the alteration in point of form, which is the conſequence of reducing the foot to its natural ſtate, but more by the abſolute increaſe of ſize in the whole limb during the progreſs of the cure. To ſhow what this has been, I ſubjoin an accurate meaſurement of the two caſts, and have marked with a dotted line on Fig. 1, plate 6, the direction in which theſe meaſures were taken, viz.

| | *Diseased Foot.* | *Restored Foot.* |
|---|---|---|
| Length of the foot, | 6 $\frac{1}{4}$ inches, | 7 $\frac{3}{4}$ inches |
| Breadth of the foot at the toes, | 3 inches, | 3 $\frac{1}{2}$ inches |
| Circumference at the toes, | 7 $\frac{1}{2}$ inches, | 8 $\frac{1}{2}$ inches |
| Over the inſtep and round the heel, | 9 inches, | 10 $\frac{1}{4}$ inches |
| Smalleſt part of the leg, | 5 $\frac{1}{2}$ inches, | 6 $\frac{1}{8}$ inches |
| Largeſt part of the leg, | 8 $\frac{1}{2}$ inches, | 9 $\frac{1}{2}$ inches |

Extraordinary as theſe circumſtances may ſeem, as the models were taken in the preſence of Mr. H. and other perſons equally reſpectable, and as they are ſtill in my poſſeſſion, it will always be eaſy to prove that theſe circumſtances are incontrovertible facts. As the reduction of the foot to this ſtate is the moſt ſtriking part of this caſe, it was my intention to ſend the hiſtory of it at this period; but as other avocations have prevented me from doing ſo, I have an opportunity now to add, that though ſo much alteration in point of form cannot take place ſince the period when the laſt model was taken as did before that time, yet minuter alterations have taken place ſo faſt, that I am now warranted in ſaying, this foot will be perfectly reſtored to its natural form, and that no one circumſtance will remain to ſhow that it has been diſtorted. I might perhaps be juſtified in ſaying as much of the reſtoration of its natural powers; but as I would avoid the imputation of raſhly advancing too much, I ſhall content myſelf with ſtating the fact, that ſhe has lately been able to walk three miles without inconvenience; and when it is remembered, that ten months ago ſhe could not go ſo many yards without aſſiſtance, and as ſhe is mending rapidly every day, I ſhall leave every one to form his own concluſions on the ſubject.

I have ſaid nothing of the left foot; for, although, if it had been the only one diſeaſed, it would have been an object of importance, it was but very little diſtorted in compariſon with the other, and it is now ſufficient to obſerve it is equally recovered; the tendency to a diſtorted ſpine too, in conſequence of the alteration alteration in the legs, has difappeared without requiring any affiftance, though if the diftortion of the legs and feet had continued, it is certain that very ferious incurvation of the fpine would have taken place.

As I wifh to avoid every imputation of exaggerating any circumftance of this cafe, it is neceffary to add, that I tranfmitted the above Narrative to Briftol, and have received the following letter from Mr. H. in reply.

"To Mr. SHELDRAKE, No. 50, Strand.

"Dear Sir,

"I have forwarded by the mail coach of this day, your Narrative of my daughter's cafe, which I have perufed with the moft affectionate intereft, and confirm in its fulleft extent, heartily wifhing its publication may excite general attention to your important and invaluable difcovery. I congratulate you and myfelf that this cafe, fo happily treated hitherto, and promifing complete fuccefs in the event, will eftablifh your claim to the approbation and applaufe of all mankind. I am extremely concerned that this wonderful cafe cannot be farther confirmed by the teftimony of that worthy gentleman* who was the caufe of my placing the child under your care; his profeffional character, no doubt, would have had great weight; he had feen the firft and fecond models, and, on that proof of advantage gained, encouraged me to hope the cure would be complete; had he lived to have feen the laft, I am convinced he would have been happy to have had this opportunity of offering this public acknowledgment of your merit, in conjunction with, "Dear Sir,

"Your obedient friend and fervant,

*Briftol*, 14 *April*, 1801. "JOHN HAYTHORNE.

"P. S. I beg you will refer any perfon defirous of feeing the models in this part of the kingdom to me; and, in cafe another is taken for yourfelf, that I may have one alfo."

If the above cafe was an anomalous one, in which the effect of fome lucky experiment had proved beneficial to the patient, I might now take leave of it with thofe fenfations that naturally will arife from the confcioufnefs of having been ferviceable to others; for as no fituation can be more powerfully contrafted than that of this patient, ten months ago, when fhe was almoft helplefs, and had no other profpect than that of increas-ing

* The late Mr. Townfend, furgeon, of Briftol, who is recently deceafed.

ing debility and its confequences, with that in which fhe is at prefent, almoft reftored to the full ufe of her powers, with life and all its profpects opening before her; fo the reflection of having produced the alteration would be fufficiently gratifying: But as this is one very ftrong example of a very numerous clafs of difeafes, few cafes of which have been radically or even palliatively cured, and as the fuccefs in this cafe was the confequence of an accurate inveftigation of the alteration produced in the parts by whatever caufed the difeafe, and a minute and laborious application of the means requifite to reftore thofe parts to their natural ftate, and as the fame principles, if fkilfully adapted and fedulloufly applied to the many varieties of the fame difeafe, will, in all probability, prove equally fuccefsful as it has done in this, I truft I fhall be excufed for adding a few obfervations on the fubject.

We continually hear of what are called contractions of the limbs, arifing, in all probability, from many different caufes; connected, it is equally probable, with very different ftates of the parts: yet the opinions of profeffional men are not fo far fettled as to point out any general fyftem upon which they may be treated with a probability of fuccefs; while the empiric, who perhaps has known fome benefit produced by fome remedy in one cafe, boldly applies the fame remedy in all cafes that he he is pleafed to call by the fame name, and thus goes on, promifing much good, and doing no little mifchief, till he is at laft funk in oblivion. It is in order to refcue the fubject from this ftate of confufion that thefe obfervations are written; and I therefore hope, if they fhould not be fuccefsful, the attempt will be excufed, and fome more fortunate and more able hand be prompted to do juftice to the fubject.

What is a contracted limb? To fome this queftion may feem unneceffary; but as I wifh to define, accurately, the data upon which I mean to argue, I fhall be excufed for obferving, that by the term contracted limb I underftand *only* that which, by the fpafmodic action of one or more mufcles, or by the lofs of fubftance or alteration in the natural texture of fome mufcles or tendons, is drawn into and firmly fixed in an unnatural pofition. Of this clafs of difeafes, the cramp and its confequences may be mentioned as caufes much in point.

When, in confequence of exercife unufually violent, and continued for an uncommon length of time, the legs become fatigued, and the patient lies down to reft without properly guarding himfelf from the effects of cold, the gaftrocnemii mufcles fuddenly and violently contract themfelves, and forcibly draw the feet into a peculiar pofition, in which they become fixed, and this is attended with excruciating pain to the fufferer, which is much increafed by any forcible attempts to reftore them them to their natural pofition. It is eafy to fee, that if this effect was permitted to continue for a long time, the motion of the feet muft ceafe; the pain in the contracted parts would diminifh as they become accuftomed to their fituation, and, at laft, would totally ceafe, except when any attempts were made to force them into action. It is likewife eafy to fee, that if this effect was continued for a longer time, fuch an alteration might take place in the organization of the contracted mufcles or tendons, as would conftitute an irremediable difeafe. The rational mode of curing this clafs of difeafes in their early ftages, and attempting to cure them in their more advanced ones, would be the ufe of the warm bath and fuch applications as can relax the parts, and mechanical applications or efforts to bring thofe parts into action in proportion as they become relaxed; but it is extremely probable, that if a pofitive alteration had taken place in the ftructure of the contracted parts, that this fyftem of treatment would produce no benefit whatever; that if incautioufly practifed it would produce much pain; and if by misfounded fortitude in the patient, or imprudent perfeverance in the operator, it was continued to excefs, great inflammation in the parts might take place, and very ferious confequences might enfue, without the poffibility of obtaining any advantage to compenfate the rifk.

Of the fuccefs of this mode of treatment, when it is fuccefsful, it cannot be fuppofed that I fhould know much, for it is not connected with my practice, and patients who have been relieved by it, need to feek no farther affiftance; of its failure in thofe cafes in which I have faid it is very unlikely, if not abfolutely impoffible, that fuch treatment fhould fucceed, I have much information; and I muft add to this, that I have no knowledge of any means by which fuch contractions as are occafioned by alteration in the ftructure of contracted parts can be cured. Of this clafs I fhall briefly mention two cafes out of the many that have fallen under my obfervation.

A gentleman in India went to fleep very imprudently in an expofed fituation; he waked in excruciating pain from the cramp; the flexor mufcles of the thigh and leg were fo contracted, as to bend the leg into the pofition it ufually is when we fit down, and in this pofition it was permanently fixed. All attempts to remove the contraction were ineffectual; he returned to England, and fought for affiftance here without fuccefs, and at laft applied to me. As every thing that could be done under the idea of relaxing, &c. had been tried, and as it appeared to me to be a cafe of permanent incurable contraction, I was not willing to make any attempt, but advifed that he fhould try, by means of an affiftant, whether the parts could be brought to act in any manner; thefe attempts, however, were attended attended with fuch excruciating pain, that they were foon difcontinued, and the patient remains in the fame ftate as when I faw him firft.

In the year 1795, I was called to the fon of a gentleman in Ireland; there was a general tendency to contraction in all the flexor mufcles of the thighs, legs, and feet; when he lay down, his knees were drawn upwards, and his toes pointed ftrait forwards; when he attempted to walk, it was only by holding a table or the chairs, and upon his toes, with his knees bent. The warm and vapour baths, electricity, fomentations, and relaxing applications had long been tried, it was faid, with good effect, and I was afked to contrive fuch inftruments as would extend and keep the limbs extended in proportion as the applications that were ufed would enable the parts to bear extenfion. I did fo, and the plan was followed with much perfeverance, and for a great length of time; but at laft all the remedies were thrown afide, as no benefit was derived from them; and if the patient be ftill living, I conclude he is in the fame condition as when I firft faw him.

By thefe and many other cafes that have fallen under my obfervation, I am authorifed to fay, that although the ufe of relaxing applications may cure fome cafes of contraction (adhering to the definition I have given of the term) yet, in general, they are ineffectual, and a fuccefsful method of curing thofe difeafes is ftill a defideratum in furgery.

To thofe limbs which, like that of Mifs H. are fixed in one pofition, and which are not diftorted by contraction, in the fenfe I have ufed that word, I would apply the term rigid, as indicating their condition, without fpecifying the caufe of it; diftorted limbs of this kind may be occafioned by many different caufes, among the moft frequent of which are the following, viz. Luxation of joints, &c. which by weakening the connecting ligaments and particular mufcles, alter the pofition, and derange the natural action of the limb, and thus throw it into an improper fituation, in which, at laft, for want of proper action, it becomes permanently fixed. In confequence of palfy, when, upon the removal of that difeafe, due care is not taken to place the limb in that fituation which is moft favourable to enable it to regain its natural powers; and many others, which as their effects are exactly the fame, it is needlefs to particularife here.

The capacity for mufcular motion in any limb depends upon the exact proportion of the power of the flexor and extenfor mufcles to each other, by which means it is enabled to perform its functions with propriety; of courfe, by whatever means the powers of thofe two fets of mufcles are rendered unequal, derangement of its action, and deformity in its fhape, muft muſt be the conſequence; that inequality, once eſtabliſhed, will continue to increaſe till the action of one ſet is deſtroyed, and that of the other will ceaſe of courſe; the limb will be motionleſs, loſe its flexibility, and diminiſh in ſize, its ſenſibility will become latent, and, at length, the whole limb be reduced as near to a lifeleſs ſtate as a part of the living animal can be. Such was the condition of Miſs H's foot; ſuch is the condition of many limbs called *contracted*, which are improperly treated, and finally abandoned in deſpair; when, by a different mode of treatment, they might be reſtored to their priſtine vigour.

As this is a point of much importance, I ſhall perhaps be excuſed for proceeding a little farther upon it. If this diſeaſe is called contraction, and it is treated with what are called emollient or relaxing applications, what will be the conſequence? The original cauſe of the diſeaſe is debility in particular muſcles; and it certainly is not eaſy to conceive how the application of relaxing remedies to parts which have loſt their power of acting from mere debility, can reſtore thoſe parts to their natural ſtate; it is eaſy to underſtand that the application of theſe remedies muſt be, at beſt, uſeleſs, when adminiſtered in every poſſible form, if they did not accelerate the progreſs of the diſeaſe; thus, by calling that diſeaſe contraction which in fact was debility, a method of treatment was adopted which was not adapted to the nature of the diſeaſe; and when it is conſidered how many ſimilar caſes there are which are treated upon the ſame principles, and with preciſely the ſame reſult, this attempt to place the ſubject in its proper point of view may not be found unneceſſary to future ſucceſs.

The leading principle upon which I acted in this caſe, and which, I believe, will prove ſucceſsful, if ſkilfully applied, in every other that is not in its nature incurable, was to reſtore the powers of thoſe parts which had loſt them as the diſtortion came on, from a perſuaſion that, if this could be done, the form and action of the whole limb would be reſtored of courſe. I am aware that this has been attempted by electricity and other means, though not with ſucceſs, I therefore merely mention the circumſtance, as it might ſeem invidious to enquire minutely into the cauſes of failure when thoſe means have been employed.

I had learned, in the ſchool of Mr. Hunter, that muſcular motion, artificially excited, would in time produce or reſtore muſcular power; to the diſcovery of the principle on which theſe diſeaſes ſhould be treated, then, I have no claim; but the merit I am entitled to, if it deſerves the name, is that of having contrived the means of reducing this principle to practice, in in the cure of a very numerous clafs of difeafes, and to any degree that may be required.

This cafe of Mifs H. has been called paralytic; if it was fo, then is this method to be called a cure for palfy; but there is abundant reafon to believe that if palfy was the primary caufe of this debility, and its confequent diftortion, *that* palfy had long been removed, and all the effects I faw were merely the effects of debility; it is not a little fingular that the fenfibility of which this leg had been fo long and fo compleatly deprived, as to give rife to a notion that it laboured under the influence of palfy, fhould be fo compleatly reftored in a few days, that much attention was requifite during the progrefs of the cure to prevent its becoming exceffive; the increafe of fize too, notwithftanding the conftant application of the bandages, &c. neceffary to effect the cure is fo very remarkable, that it requires every voucher of its authenticity to be carefully examined before implicit credit will be given to the account of it.

If any fhould object that I go too far in arguing, from the cure of this cafe, that every other cafe of the fame defcription may as certainly be cured, I fhall beg leave to obferve that the greateft certainly includes all the lefs, and many cafes of the fame kind, but lefs in degree, I have had under my care, fucceeding equally with them all; I believe too it is fair induction to fay, that if there are worfe cafes of the fame kind, it is very probable, nay almoft certain, that the fame means, if duly proportioned, and feduloufly applied, will certainly cure every cafe, however bad it may be, till it arrives at that point which oppofes fome phyfical impoffibility to prevent our fuccefs.

At what period that impoffibility may take place it is not eafy to fay, though it certainly does not fo early as it has been fuppofed; and it is extremely probable it will be much later in life, and much lefs frequently, than many will imagine. As it is of confequence that this doctrine fhould be firmly eftablifhed, I fhall perhaps be excufed for fupporting it by referring to a quarter from whence information on this fubject might not be expected.

A late author,* in defcribing the extraordinary mortification of a devotee, fays, "The compleat term of his firft penance being expired, the next he undertook was to hold his hands, locked in each other, over his head, the fingers of one hand dividing thofe of the other, for the fame fpace of twelve years. —When I firft faw him at this place, in the year 1783, he rode on a piebald Tangun horfe from Bootan, and wore a fatin embroidered

* Turner's Embaffy to the court of the Tefhoo Lama.

embroidered drefs given to him by Tefhoo Lama, of which he was not a little vain. He was robuft and hale, and his complexion, contrafted with a long bufhy black beard, appeared really florid. I do not fuppofe that he was then forty years of age. Two Gofeins attended him, and affifted him in mounting and alighting from his horfe; indeed, he was indebted to them for the affiftance of their hands on every occafion; his own being fixed and immovable, in the pofition in which he had fixed them, were of courfe perfectly ufelefs.

" *The circulation of the blood feemed to have forfaken his arms, they were withered, void of fenfation, and inflexible; yet he fpoke to me with confidence of recovering the ufe of them, and mentioned his intention to take them down the following year, when the term of his penance would expire.*

" Other Gofeins affured me, *though I could not help doubting the fact*, that it is practicable to reftore withered limbs, thus circumftanced, to perfect ufe. This is effected, they fay, though not without great labour and fome pain, by means of long continued friction, before a large fire, with a certain ointment which they compound."

The authority of thefe Gofeins is not to be very highly eftimated as to the furgical treatment of the difeafe, though they may fafely be admitted as evidences to the fact mentioned by Capt. Turner. The appearance of the man fo crippled is not mentioned as unique, it is ftated, and indeed known, to be the regular practice of a certain defcription of perfons who profefs the fame religion; and though it is a fpecies of fuperftition, it is not of that gloomy kind which has led many to deftruction, but, with the calmnefs of miftaken philofophy, that man willingly rendered his arms ufelefs for twelve years, from a belief that the facrifice will be grateful to his Deity, and a conviction that at the expiration of the time mentioned in his vow, he *can take them down again, and recover the perfect ufe of them.* His countrymen likewife believe this, and ftate the means by which it may be done, not as a matter of doubt, but as of the moft abfolute certainty, and, undoubtedly, not without fufficient reafon.

There are no practices more antient than the fuperftitions of India; they have been invariably followed, without the leaft deviation, for a feries of ages, of which perhaps we have no adequate idea; and it refults from our knowledge of the facts mentioned by Capt. Turner, that perfons at the prefent time do what has been regularly done by their countrymen for time immemorial, render their arms ufelefs for the term of twelve years, and at the end of that term reftore them to their original perfect ftate. Capt. T. doubted the fact, becaufe he had no knowledge of any thing similar, yet the terms in which he describes the man's *arms*, if the word *leg* was substituted for it, would be very descriptive of Miss H's leg; the cure of this is within our knowledge, and justifies us in believing what is related of this man by Capt. T. which again will justify us in concluding that many persons with the same kind of defects, and much farther advanced than that of Miss H. is, may be perfectly cured by the same means that were employed in curing her.

Mr. H. in his letter calls this a wonderful case; others, who are not equally affected by it, may call it an extraordinary one. As I do not wish for the reputation of working miracles, and am desirous to subject what seems to be extraordinary to the judgement of those who are competent to decide upon it, I shall state what appears to me to be the progress of a limb in the condition this lady's was, from the healthy to the diseased state, and on its return to the healthy state again.

In consequence of weakness, from whatever cause, the extensor muscles of the foot were incapable of performing their functions properly; the contractile action of the flexors was of course increased, and the foot thus drawn into an unnatural position; this derangement, deformity, and rigidity increased till she had no power to use the leg; in this state it diminished in size for want of action, and lost its sensibility from the same cause. In this state it was called a *contracted leg and foot*, and every application made to it, instead of curing, only increased its debility, and at length it became useless.

Having formed a different opinion of it, I avoided all the farrago of emollients, &c. as I would have done the ointment of the Goseins, and confined my treatment to the application of bandages, consisting of springs so adapted as to imitate the action of every muscle whose power was deficient, and by altering, adapting, and modifying these applications, so as to produce the action of every muscle in its turn, I proceeded by degrees till the foot was reduced to its natural form, and so much of its natural powers restored, as leaves a well-founded hope that every the least appearance of disease will be exterminated.

The physical alteration in the state of the parts seems to have taken place in the following manner: By applying a spring to draw the foot towards the same position in which it would be placed by the action of any particular muscle, that muscle is brought to act though it had not before the power to do so; and, in consequence of this action, the general circulation is increased through the whole limb; the consequence of this increased circulation is enlargement of the vessels through which which the fluids circulate, and of course an increase of size and sensibility in the whole limb. The application to produce the action of every muscle must be changed and varied according to the feelings of the patient; but, however it may be necessary to vary these applications, the general action of the limb is perpetually kept up, and if duly regulated, the restoration of feeling, size, shape, and, finally, the natural power of action is completely and permanently restored.

